# Ultrasonographic features associated with previous torsion and the impact of surgery in managing neonatal ovarian cysts: a 20-year single-centre retrospective study

**DOI:** 10.1007/s00383-023-05458-2

**Published:** 2023-04-24

**Authors:** Athanasios Tyraskis, Joseph Davidson, Jennifer Billington, Simon Blackburn, Joseph Curry, Dhanya Mullassery, Stefano Giuliani, Simon Eaton, Kate Cross, Paolo De Coppi

**Affiliations:** 1 Department of Paediatric Surgery, King’s College Hospital, London, UK; 2 Stem Cells and Regenerative Medicine, Developmental Biology and Cancer Programme, UCL Great Ormond Street Institute of Child Health, 30 Guilford Street, London, WC1N 1EH UK; 3 Neonatal & Paediatric Surgery and Biomedical Research Center, Great Ormond Street Hospital, London, UK

**Keywords:** Neonatal ovarian cysts, Ovarian torsion, Antenatal ultrasound, Complex ovarian cyst

## Abstract

**Purpose:**

To identify markers of previous ovarian torsion and outline the outcomes according to US appearance and operative management.

**Methods:**

A retrospective single-centre review of neonatal ovarian cysts from January 2000 to January 2020. Data on postnatal cyst size and sonographic features and operative treatment were co-related with outcomes of ovarian loss and histology.

**Results:**

77 females were included with 22 simple and 56 complex cysts, one patient had bilateral cysts. 9/22 (41%) simple cysts regressed spontaneously in a median of 13 weeks (8–17). Complex cysts regressed spontaneously less frequently, 7/56(12%, *P* = 0.01), in 13 weeks (7–39). 38/56 (68%) complex and 12/22 (55%) simple cysts were treated operatively. 21/22 (95%) ovaries with initially simple cyst were salvaged compared to 20/56(36%) with initially complex cyst (*P* < 0.001). A fluid-debris level in 23/26 complex cysts was most associated with ovarian loss (*P* = 0.0006). Presence of viable ovarian stromal tissue was seen in 8/20 (40%) excised specimens during ovarian sparing procedures and in 5/30 (17%) oophorectomies for necrotic appearing ovaries.

**Conclusions:**

Fluid-debris level on US is significantly associated with ovarian loss likely due to previous torsion. Simple cysts are viable and often regress spontaneously. The finding of viable ovarian stromal tissue in resected specimens supports attempting ovarian preservation wherever possible.

## Introduction

Cysts of ovarian origin are the most common intra-abdominal cyst detected in females perinatally [1]. These usually follicular cysts originate in foetal life as a response to maternal and placental oestrogens and gonadotrophins [2]. Postnatally hormonal levels decline in the neonates circulation and this is associated with a decrease in size in many antenatally diagnosed cysts, especially when they are simple in appearance [3]. There is a well-documented association of larger cysts with ovarian torsion in the prenatal period and that risk is likely to remain in the early postnatal period [3].

Debate remains on the optimal management of patients with larger ovarian cysts postnatally. This is due to balancing the risk of torsion postnatally in cysts that are likely to spontaneously regress when simple, or possibly have already torted when complex. It is unclear if information from postnatal ultrasound (US) scans can aid in understanding which cysts stand to benefit most from early surgical management.

## Method

This is a single-centre retrospective study of patients of less than 1 year of age presenting to Great Ormond Street Hospital between January 2000 and January 2020. This study was registered as an audit approved by the Clinical Audit and Safety Department of Great Ormond Street Hospital (approval number: 1524).

We included all cases with intra-abdominal cysts of ovarian origin and excluded cases that were subsequently determined to have cysts of other origins. We gathered all available data on cyst dimensions and appearance from all postnatal ultrasound (US) scans. Patients were followed up until cyst resolution, surgery, or multiple scans showed the absence of an ovary on the suspected side of the cyst – indicating likely ovarian loss. Ovarian salvage was defined as bilaterally normal ovaries at discharge. Where available, histology results were also reviewed. Primary outcome was having two viable ovaries at the time of discharge and secondary outcomes were surgery and review of any tissues sent for histological analysis. We aimed to find markers of previous ovarian loss as well to outline outcomes according to US findings and operative or non-operative management.

Simple cysts were defined as a thin-walled cyst with anechoic contents, and complex cysts included those with internal septations, debris, fluid levels, or appearance of cystic and solid component. Ovarian loss in patients with ovarian cysts may occur due to torsion, and for the purpose of this study was defined as: a necrotic ovary at the time of surgery, or a complex cyst which regressed without any identifiable ovarian tissue on the ipsilateral side on more than one US scans post cyst regression. Resolution was defined as resorption of the cyst with two identifiable ovaries on US.

Continuous data were reported as median and interquartile range and tested using a Mann–Whitney U test. Two-tailed Fisher’s exact tests were used to test for significant differences between the different size groups, GraphPad Prism (Version 6)^**®**^ was used for this statistical analysis. 95% level of confidence was defined as significant. Finally, a 95% confidence interval of proportion was calculated using the GraphPad Quick Calcs online software. A *P* value of < 0.05 was considered significant.

## Results

### Patients and demographic data

A total of 77 females were eligible for inclusion in our study who had a total of 78 ovaries with significant cysts – one patient having bilateral cysts. 56 (73%) of those patients noted an antenatal diagnosis prompting referral and the remaining were found in the neonatal period. Only one patient presented acutely with symptoms – they had a complex cyst with abdominal pain and subsequently underwent surgery which found a necrotic cyst with no ovarian tissue present. All remaining patients had asymptomatic cysts.

The median gestational age at birth was 39 weeks (IQR 37–40 weeks) and median birth weight was 3.3 kg (IQR 3.0–3.8 kg). Data on gestational age at birth and birth weight was not available for 10 and 27 patients respectively. 1 patient’s care was transferred before a final outcome was reached and 3 patients were lost to follow-up.

### Ultrasonographic features

The median age of the first postnatal US scan was 1 week (IQR 0.4–2.8 weeks). In 48 (62%) patients the cyst was identified on the right, in 28 (36%) on the left, and in 1 (1%) they were bilateral (*P* = 0.002). On the initial postnatal scan 22 (28%) of the cysts were simple and 56 (72%) were complex (*P* = 0.0001). In addition, one patient had an associated comorbidity of congenital adrenal hyperplasia and interestingly had a normal US initially at 6 weeks with a simple cyst developing in the first 4 months of life and growing to a greatest diameter of 63 mm and subsequently regressing by 9 months of age.

Outcomes of simple and complex cysts are highlighted in Table 1. They were similar in terms of cyst size and demographic data of the patients but had significantly different outcomes. 21/22 (95%) of patients with simple cysts had who viable ovaries at discharge compared to 20/56 (36%) of those with complex cysts (*P* = 0.0001). The proportion of spontaneous regression in simple cysts was 9/22 (41%) compared to only 7/56 (13%) of complex cyst (*P* = 0.011) at a median time of 8 weeks (IQR 5–27). Proportions of operatively managed complex cysts were 38/56 (68%), similar to the simple cysts 12/22 (55%) (*P* = 0.3). Median age at operation for simple cysts was 7 weeks (IQR 3–12) which was not significantly different from the complex group at 9 weeks (IQR 5–22) (*P* = 0.31).

**Table 1:** Table comparing features and outcomes of simple cysts compared to complex cysts

	Simple	Complex
Total Patients	22	56
Lost to follow-up or transfer of care (%)	1 (5)	3 (5)
Median GA at birth in weeks (IQR)	38 (36–39)	39 (38–40)
Median birth weight in kg (IQR)	3.3 (3.2–3.9)	3.3 (2.9–3.7)
Median greatest cyst diameter on first postnatal scan in mm (IQR)	51 (38–73)	49 (40–55)
Operative management (%)	12 (55)	38 (68)
Spontaneous regression with both ovaries present (%)	9 (41)	7 (13)
Bilateral normal ovaries at discharge	21 (95)	20 (36)
Ovarian loss Necrotic ovary confirmed at surgery Cyst regressed with only one ovary detectable	1 (5) 1 (5) 0	36 (64) 28 (50) 8 (14)

US features that were associated with ovarian loss are outlined in Table 2. Within the complex group, the presence of a fluid-debris level was significantly more common in cases with ovarian loss (*P* = 0.0006) and it had a sensitivity of 64% and specificity of 85% for ovarian loss in our cohort.

**Table 2:** US features and proportion of ovarian loss

	Number of patients	Spontaneous regression (%)	Ovarian loss (%)
Simple	22	9 (41)	1 (5)
Complex—all	56	7 (13)	36 (64)
Complex—Features not described	11	1 (9)	3 (27)
Complex—Septa only	10	1 (10)	3 (30)
Complex—Fluid-debris level	26*	3 (12)	23 (88)
Complex—Appearance of part solid component	14*	3 (21)	11 (71)

*5 patients with fluid-debris levels also had appearances of part solid components and are included in both groups

### Cyst size

**Fig. 1:**
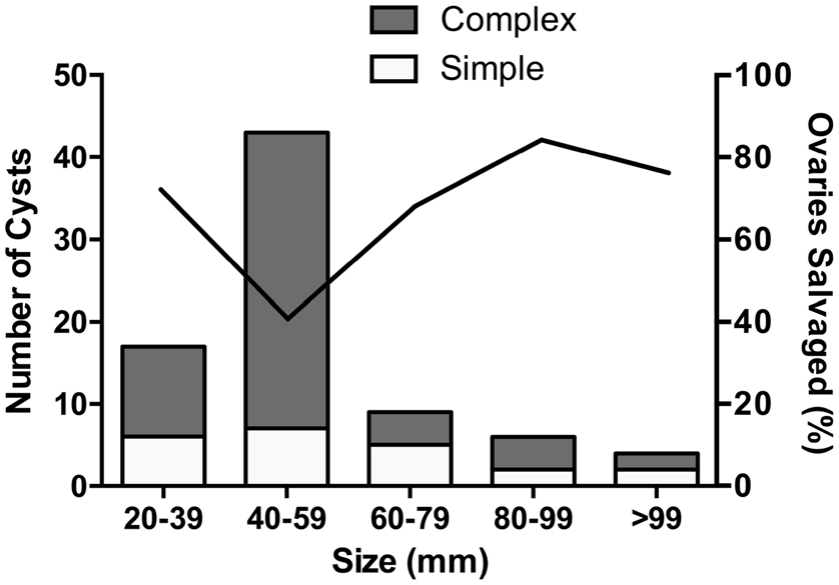
Figure of cysts by greatest diameter on first postnatal scan showing simple and complex cysts and subsequent risk of ovarian loss

Figure 1 shows the number of cysts by size as well as the proportion of ovaries salvaged with normal ovaries bilaterally at discharge. The highest risk group was 40-59 mm of which 17/42 (40%) ovaries were normal at discharge. Interestingly, larger cysts over 60 mm had higher salvage rates. 14/19 (74%) of those 60 mm and over were salvaged compared to 27/59 (46%) of cysts up to 59 mm—(*P* = 0.039). The groups had similar age at operation – median age was 9.5 weeks (IQR 5–22) in the 40-59 mm group compared to 7 (IQR 2–11) in cysts 60 and over— *P* = 0.28.

We also investigated if the initial change in size between the first two US were useful in predicting a viable ovary. This was possible for 49 (64%) of the patients. An increase in size was uncommon and was seen in only 5/49 (10%) patients. 20/49 (41%) remained within 10% of the initial size and the remaining 24/49 (49%) decreased in size. Cysts that were decreasing in size had higher proportion of ovarian salvage 16/24 (67%) compared to 11/25 (44%) remaining stable or increasing in size, although this failed to reach statistical significance (*P* = 0.15). 4/5 (80%) of those with an increase in size were salvaged.

### Surgical management

50 (65%) patients were treated surgically. 31 (62%) patients underwent laparoscopic interventions, 10 (20%) underwent laparotomy, 6 (12%) patients had laparoscopic converted to open operations, 1 (2%) patient underwent a percutaneous aspiration, and in 2 (4%) patients operative data were missing. 20 (40%) aimed to have ovarian sparing procedures (aspiration, deroofing, cystectomy, or marsupialization) and 30 (60%) underwent resection of necrotic appearing cysts. One underwent a laparoscopy and only small follicular cysts were seen (thus the previous cyst had regressed). In three patients who underwent surgery 180, 360, and 720 degree twisting was noted without any ischaemia and deroofing, cystectomy and marsupialization were used to preserve the viable ovary.

We co-related the histological findings with the operative technique used and found that it was difficult not to remove ovarian tissue with cyst wall. 8/20 (40%) of ovarian sparing procedures included ovarian stoma or oocytes in the histological specimen. Greater accuracy was achieved when resecting the 30 cysts that appeared torted and necrotic as 25/30 (83%) confirmed no viable ovarian tissue present. Of those, 6/30 (20%) were autoamputated, all of which had no viable ovarian tissue. However, in 5 (17%) patients with apparent torsion who underwent oophorectomy some viable ovarian tissue. 1 patient had a resection of a viable ovary without necrosis (that prior US showed a mass that seemed to have a solid component) and was a large haemorrhagic cyst without a detectible ovary separate to this as it had stretched ovarian stroma from the cyst (confirmed on histology). No evidence of malignancy was seen on any specimen.

We also correlated the impact of a laparoscopic intervention compared to those requiring laparotomy. 2 patients with uncertain modality of operation and 1 patient who underwent aspiration were excluded from this analysis. Of the laparoscopically treated group 16/31 (52%) underwent oophorectomy and 15/31 (48%) underwent ovarian sparing procedures. There was a higher proportion of patients who underwent oophorectomy in the open group 12/16 (75%) although this was not statistically significant (*P* = 0.21). 2/16 (13%) oophorectomies performed laparoscopically had some viable ovarian tissue compared to 3/11 (27%) performed open (*P* = 0.37). Regarding ovarian sparing procedures, 6/15 (40%) of those performed laparoscopically had some viable ovarian tissue in the histology specimen compared to all 4/4 (100%) of those performed open (*P* = 0.09). Importantly, in ovarian sparing procedures the presence of some ovarian tissue is likely a minor amount of the respective ovary as it will be tissue stretched out by the cyst resected at the time of partial cyst wall resection.

## Discussion

The greatest challenge in managing neonates with ovarian cysts is determining when it is beneficial to operate. Necrotic cysts that may be reabsorbed may have no benefit but only risk from operative resection, and viable cysts that are at potential risk of torsion are also the most likely to regress spontaneously. Our study found that fluid-debris levels are a useful indicator when determining if a neonatal ovarian cyst may already be necrotic. We also highlighted some of the risks with operative management such as removing viable ovarian tissue whilst also observing that non-ischaemic twisting of an ovarian cyst may also occur.

Malignant ovarian lesions under the first year of life are exceptionally rare, and cases in the reported literature are few and far between. Cass et al. reported one immature teratoma in a 15 years series of 102 ovarian masses and Panteli et al. detected none in their 40 patient series [4, 5]. Three further cases of sex cord stromal tumours in the first year of life have been reported, all of whom had evidence of precocious puberty. One case was confirmed to be present at birth and one further was postulated due to high levels of testosterone and anti-Mullerian hormone [6–9]. Thus, antenatal or neonatal simple cysts with no clinical or biochemical evidence of hormonal abnormalities have an exceptionally low risk of malignancy regardless of size.

When considering the size of the cyst, in our previous work we noted that cyst size was associated with the risk of torsion and chance of spontaneous resolution. Specifically, we found 40 mm of greatest diameter in the antenatal period to be associated with an increased risk of torsion and cyst smaller than 40 mm more likely to regress spontaneously [2, 3]. In this study we also found that larger cysts of 60 mm had higher rates of ovarian salvage. Several factors may be able to account for this finding. Firstly, a smaller proportion of complex cysts were present in the larger group (53% vs. 80%) thus there may be confounding factors. A second possibility is that a torted ovarian cysts is less likely to have viable epithelial cells that continue respond to maternal hormones and grow further and reach larger sizes. Finally, when a cyst grows to vary large sizes its mobility within the pelvis and abdomen may be restricted due to less space in the intraperitoneal cavity relatively to the cyst’s size.

The timing of torsion and ovarian loss in our cohort is not obvious but the clinical evidence supports the majority of cases occurring antenatally. Only one patient presented symptomatic postnatally. However, torsion may have an atypical presentation in a neonate and its detection may be difficult. We did identify that 3/77 (4%) of the cases had viable but twisted ovaries at the time of operation without symptoms.

The intrauterine environment promotes growth of follicular cysts due to high levels of follicle-stimulating hormone (FSH), estrogens, human chorionic gonadotropin (HCG), and luteinizing hormone (LH). Postnatally all of these hormones decline leading to the spontaneous regression of most follicular cysts. In our series the median time to spontaneous regression was 8 weeks. Given some complex cysts regress with no remaining ipsilateral ovary, previously torted ovarian cysts that may self-resolve will not benefit from surgery. However, persistent cysts that there may also be diagnostic uncertainty (e.g. of an enteric duplication), or ‘wandering’ complex cysts that may have autoamputated may represent indication for laparoscopy. Previously reported cases of a “wandering” ovarian cysts leading to adhesional intestinal obstruction have been reviewed by Jeanty et al. and found 9 cases of adhesional obstruction in the reported literature that represented 3% of the cases of neonatal ovarian cysts they reviewed [10].

Regarding operative management, the message has become clear over the years and, care must be taken to preserve as much ovarian tissue as possible. Overwhelming evidence supporting ovarian detorsion for the management of ovarian torsion and avoiding oophorectomy in paediatric patients [11]. Long term analysis of the treatment of ovarian torsion revealed that ovaries undergoing detorsion and left in place preserved their function [12]. Pregnancies have occurred in patients after detorsion of an ovary both spontaneously and with harvested oocytes from previously torted ovaries. At surgery, large ovarian cysts may have a misleading appearance, due to tissue distortion stretching the ovarian cortex around the cyst. Any deroofing or cystectomy must take care to remove as little tissue as possible. Autoamputation is the only scenario where viable ovarian tissue in the cyst highly unlikely (none of 6 autoamputated cysts had any viable ovarian tissue in our series). However, in torted apparently necrotic cysts that are not autoamputated, some ovarian tissue may still be present. In our series we found 5/24 (21%) of torted necrotic appearing cysts that were not autoamputated still had some viable ovarian tissue in the histology specimen. Thus, even when viable ovarian tissue is not evident in the context of torsion, similar to how acute presentations of ovarian torsion are managed. The consensus recommendation for imaging surveillance following ovarian detorsion is an ultrasound at 3 months post-procedure but sooner if there is a concern for malignancy.

## Conclusion

Fluid-debris level in postnatal ultrasound is associated with previous ovarian torsion and necrosis. Simple cysts are viable and likely to regress spontaneously, remaining risk of torsion appears low as postnatal symptomatic torsion was rare. Surgical interventions risk resection of viable ovarian tissue and ovarian preservation should be undertaken whenever possible in both non-torted ovaries as well torted necrotic appearing ovarian cysts. Autoamputated ovarian cysts can be safely excised.

## Data Availability

The data that support the findings of this study are not openly available due to reasons of patient confidentiality, however, upon reasonable request components of data can be sought. Data are located in controlled access data storage at Great Ormond Street Hospital.
